# Relationships between Characteristics of Urban Green Land Cover and Mental Health in U.S. Metropolitan Areas

**DOI:** 10.3390/ijerph15020340

**Published:** 2018-02-14

**Authors:** Wei-Lun Tsai, Melissa R. McHale, Viniece Jennings, Oriol Marquet, J. Aaron Hipp, Yu-Fai Leung, Myron F. Floyd

**Affiliations:** 1 Department of Parks, Recreation and Tourism Management, North Carolina State University, Box 8004, Raleigh, NC 27695-8004, USA; wtsai@ncsu.edu (W.-L.T.); omarque@ncsu.edu (O.M.); jahipp@ncsu.edu (J.A.H.); leung@ncsu.edu (Y.-F.L.); 2 Natural Resource Ecology Lab, Department of Ecosystem Science and Sustainability, 1499 Campus Delivery, Colorado State University, Fort Collins, CO 80523-1499, USA; melissa.mchale@colostate.edu; 3 US Forest Service, Southern Research Station, Athens, GA 30602, USA; vjennings02@fs.fed.us; 4 Center for Geospatial Analytics, North Carolina State University, Box 7106, Raleigh, NC 27695-7106, USA

**Keywords:** green land cover, urban forests, urbanization, landscape

## Abstract

Urbanization increases risk for depression and other mental disorders. A growing body of research indicates the natural environment confers numerous psychological benefits including alleviation of mental distress. This study examined land cover types and landscape metrics in relation to mental health for 276 U.S. counties within metropolitan areas having a population of 1 million or more. County Health Rankings and Behavioral Risk and Factor Surveillance System (BRFSS) provided a measure of mental health. The 2011 National Land Cover Database (NLCD) provided data on green land cover types, from which seven landscape metrics were generated to characterize landscape patterns. Spearman’s rho correlation and stepwise logistic regression models, respectively, were employed to examine bivariate and multivariate relationships. Models were adjusted for county population and housing density, region, race, and income to account for potential confounding. Overall, individual measures of landscape patterns showed stronger associations with mental health than percent total cover alone. Greater edge contrast was associated with 3.81% lower odds of Frequent Mental Distress (FMD) (Adjusted Odd’s Ratio (AOR) = 0.9619, 95% CI = 0.9371, 0.9860). Shrubland cohesion was associated with greater odds of FMD (AOR = 1.0751, 95% CI = 1.0196, 1.1379). In addition, distance between shrubland cover was associated with greater odds of FMD (AOR = 1.0027, 95% CI = 1.0016, 1.0041). Although effect sizes were small, findings suggest different types of landscape characteristics may have different roles in improving mental health.

## 1. Introduction

**Figure 1:**
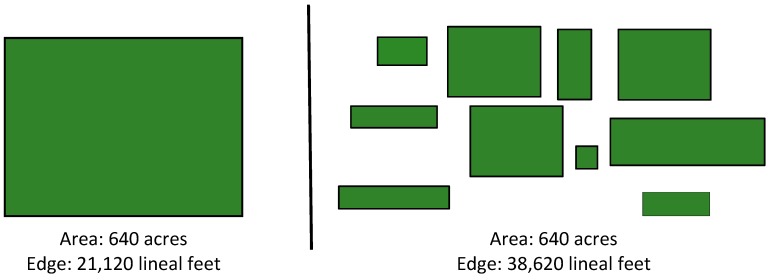
Diagram representing total forest cover, smaller patches, corridors, and increased edges (Adapted from C.E. Adams).

Urban green space has been associated with numerous health benefits. Access to public green space provides opportunities for physical activity, increased social cohesion, and improved psychological well-being [1,2,3,4,5,6]. Therefore, increased urbanization and potential loss of green vegetative cover including trees and open space in cities may hold important implications for human health [7]. Globally, more than half of the population inhabits urban areas. One common health challenge for urban inhabitants is mental distress [3,8]. Environmental exposures in urban built environments produce numerous stressors that adversely associate with mental health [9,10,11]. A meta-analysis of rural-urban differences in psychiatric disorders found that mood and anxiety disorders were more prevalent in urban areas [12]. Frequent mental distress has been linked with several health issues such as coronary heart disease and stroke [13]. Green land cover, even small and fragmented in urban areas [14], provides a range of benefits which can improve mental well-being and other aspects of preventive medicine [15]. For instance, studies correlate the presence of green spaces to fewer symptoms of depression [16], stress [17], and improved measures of well-being [18]. A twin study of effects of access to green space on self-reported depression, stress, and anxiety found significant associations for depression but none for stress and anxiety [19]. A longitudinal study in Britain explored the role of green space on residential happiness and found that people living in cities with more green space reported less mental distress and more well-being [20]. However, most of the studies only utilize total percent cover or greenness (e.g., percent tree cover and Normalized Difference Vegetation Index, respectively) [19,21,22], which mostly show the amount of green land cover and cannot provide information about specific landscape characteristics and their association with health outcomes. For example, as illustrated in Figure 1 (adapted from Adams [23]), forest cover varies in configuration and patterns. Large intact forest patches may exhibit different relationships to health and well-being outcomes than fragmented forest landscapes. Such differences could mean that some environmental attributes may better meet human psychological needs or provide better opportunities for contact with nature. For instance, forest edges have been associated with feelings of security because they fulfill the needs related to open views and enclosure as suggested by prospect refuge theory [24]. More edge density of green land cover may also imply more opportunities for people to realize mental health benefits from contact with nature in general [25], as well as nearby nature, such as street trees and gardens [14]. Moreover, different land cover types as well as landscape patterns may also have different effects on mental health [26]. Given growing interest in sustainable health promotion in cities [27], this study examined relationships between mental health and urban green land cover characteristics for 276 U.S. counties within metropolitan areas having a population of 1 million or more. To summarize, this study addresses the following hypotheses:

**Hypothesis 1.**  Specific landscape characteristics are more strongly associated with mental health than amount of green land cover alone.

**Hypothesis 2.**  Types of green land cover are associated differently with mental health.

## 2. Methods

### 2.1. Study Area

U.S. metropolitan statistical areas (MSA) with more than 1 million in population (52 MSAs) were selected and analyzed at the county level (N = 276). The 52 MSA were divided into five regions based on the US Environmental Protection Agency’s ecoregion classification: Northeast (N = 34, including States of Maine, Vermont, New Hemisphere, Connecticut, New York, New Jersey, Massachusetts, Rhode Island, Pennsylvania, Maryland, and Delaware), Southeast (N = 106, including District of Columbia and States of West Virginia, Virginia, Kentucky, North Carolina, Tennessee, South Carolina, Georgia, Alabama, Mississippi, Arkansas, Florida, and Louisiana), Midwest (N = 61, including States of Michigan, Ohio, Indiana, Illinois, Minnesota, Wisconsin, Iowa, Missouri, North Dakoda, South Dakoda, Nebraska, and Kansas), West (N = 35, including States of Montana, Wyoming, Idaho, Colorado, Utah, Nevada, Washington, Oregon, and California), and Southwest (N = 40, including States of Oklahoma, Texas, New Mexico, and Arizona).

### 2.2. Mental Health

A mental health measure, Frequent Mental Distress, was obtained from County Health Rankings and Roadmaps and was derived from the 2014 U.S. Centers for Disease Control Behavioral Risk and Factor Surveillance System (BRFSS) [28]. Specifically, Frequent Mental Distress (FMD) was defined as percent of county respondents reporting 14 to 30 mentally unhealthy days in the past 30 days using the following question: *Now thinking about your mental health, which includes stress, depression, and problems with emotions, for how many days during the past 30 days was your mental health not good?* (See Moriarty et al. [29] for detailed background on this measure). To classify counties into low and high levels of FMD, the Jenks Natural Breaks classification was employed [30]. This approach classifies observations into groups where the within-group variance is minimal and between-group variance is maximal. Thus, FMD was dichotomized using the break value to reflect low and high prevalence of FMD.

### 2.3. Land Cover

The 2011 National Land Cover Database (NLCD), a 30-m resolution raster dataset, classifies land cover into eight major types based on Anderson Level I Classification System [31] and provided measures of green land cover types (forest, shrubland, and herbaceous). Forests are areas dominated by trees with a height generally taller than 5 meters, shrubland refers to shrubs generally less than 5 meters in height and young trees at an early successional stage, and herbaceous refers to grassland or low vegetation and typically accounts for more than 75% of the cover [32]. Seven landscape metrics (percent cover, mean patch area, patch density, edge density, edge contrast index, Euclidean distance between patches, and patch cohesion index) were generated for each land cover type at the county level by FRAGSTATS 4.2 (Computer Software Program, University of Massachusetts, Amherst, MA, USA) [33]. Table 1 provides definitions for each of the seven landscape metrics. The selected metrics were chosen to characterize the patterns of green land cover based on existing theory and empirical studies that describe how natural environmental attributes potentially provide opportunities for human contact with nature or meet basic human psychological needs [34,35,36]. For instance, higher percentage of greenspace was found to associate with less stress, depression, and better mental health (percent cover) [37,38,39]. Generally having small parks but close to residential areas, provide more opportunities for urban inhabitants to use (patch size and patch cohesion index) [40]. Distance to small patches of urban greenery, such as greenspaces in urban residential areas, also supports greater frequency usage when compared to other urban greenspaces (e.g., urban parks) (patch size and Euclidean distance between patches) [41]. Likewise, availability of greenspaces increases the likelihood of walking activity (patch density) [42]. Edge features in an environmental setting provide psychological needs for human to both observe and hide (edge density and edge contrast index) [24,36].

### 2.4. Confounding Variables

Population density, housing density, median household income and race (percentage of non-White racial/ethnic groups) were measured and treated as potential confounding variables in the statistical models [43]. These measures were extracted from the U.S. Census American Community Survey [44]. Because previous research found geographic differences in mental health in the U.S. [29,45], region also served as a potential confounder. The inclusion of region also accounts for differences in landscape biogeographic features in each region.

### 2.5. Statistical Analysis

Spearman’s rho provided tests of bivariate relationships with the exception of bivariate analysis of region and FMD where one-way Analysis of Variance was used. Stepwise logistic regression was performed to select the most influential landscape variables. Land cover types and landscape metrics of all three types (21 total) were first entered into the model as a pool of candidate variables, and then the set of predictor variables for FMD were finalized by using the Akaike Information Criterion as the selection criterion with bidirectional elimination. A subsequent model provided estimates of effects of the selected landscape metrics on FMD, adjusting for ecoregion and potential confounders. Generalized Variance Inflation Factor (GVIF) values greater than two indicated concerns with multicollinearity. Regression analyses were performed in R Studio 0.98.1103 (RStudio Inc, Boston, MA, USA).

## 3. Results

**Figure 2:**
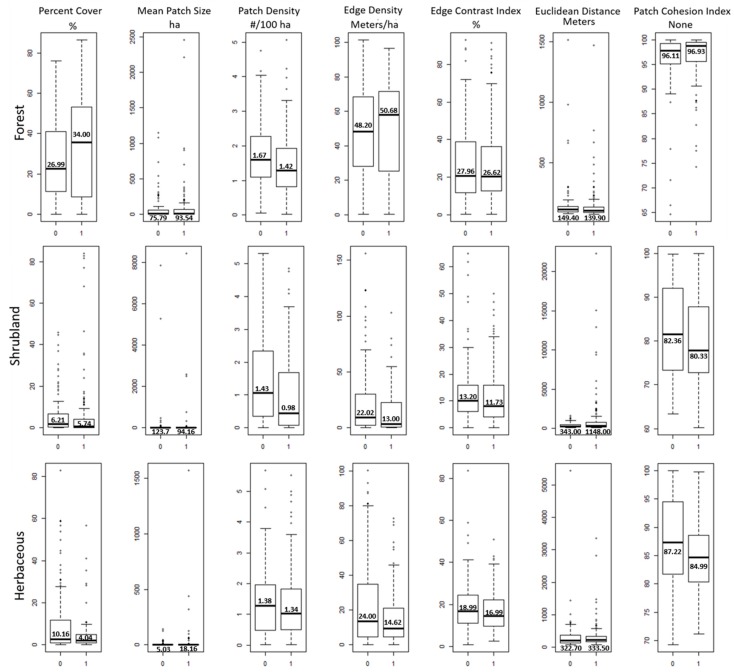
Descriptive statistics of FMD (Frequent Mental Distress) level by land cover and landscape metrics (A value of 0 on the X-axis indicates low levels of FMD (≤10%) and 1 indicates high level of FMD rate). Numerals within the plots are mean values).

Descriptive statistics for study variables are presented in Table 2. FMD values ranged from 7% to 15% with a mean of 10.76% across the 276 counties. Using the Jenks Natural Breaks approach to dichotomize FMD, the break was identified at 10%. Using this break value as the cut point, 42.75% of counties were classified as low prevalence of FMD. One hundred eighteen counties (24 western, 33 southwestern, 23 midwestern, 16 northeastern, and 22 southeastern) were considered to have low prevalence of FMD. Most of the counties in the southeastern region (84 out of 106) had higher prevalence of FMD. Mean comparisons revealed that the prevalence of higher FMD was significantly greater in the southeast region (Table 3). Mean percent forest cover, edge density, and patch cohesion of forests were greater in counties with the higher prevalence of FMD. Shrubland and herbaceous cover were higher in counties with the low levels of FMD (Figure 2). Mean patch size was larger at the high level of FMD for forest and herbaceous cover but smaller for shrubland cover. Mean patch density and edge contrast index were consistently higher for all types of green land cover at the lower levels of FMD. Mean Euclidean distance between patches of forest and herbaceous cover were similar at both FMD levels but was much greater at the high level of FMD for shrubland cover.

Among the green land cover types, forest cover was most positively associated with the prevalence of FMD, whereas shrubland and herbaceous types had more negative significant associations with FMD (Table 4). Farther distance between forest patches had the strongest negative association with the prevalence of FMD (rho = −0.18, *p* < 0.01), whereas closer distance between shrubland patches had the strongest positive association with the prevalence of FMD (rho = 0.19, *p* < 0.01). Among the confounding variables, median household income (rho = −0.12, *p* < 0.05) and region (*F* = 26.15, *p* < 0.00001) were statistically associated with FMD.

Ten landscape metrics (%forest, %shrubland, %herbaceous, edge contrast index of forest and shrubland, mean patch size of shrubland, Euclidean distance between shrubland patches, patch cohesion index of shrubland, and patch density and edge density of herbaceous) were selected as predictors for the model following stepwise regression. Percent shrubland, edge density of herbaceous cover, and housing density were dropped from model estimation due to GVIF values greater than 2. After adjusting for region and potential confounders, increased forest edge contrast (F_ECON) was associated with 3.81% lower odds high level of FMD (Adjusted Odds Ratio = 0.9619, 95% CI = 0.9371, 0.9860) (Table 5). Shrubland cohesion (more connected shrubland) was associated with greater odds of FMD (AOR = 1.0751, 95% CI = 1.0196, 1.1379). Distance between scrubland cover was also associated with greater odds of FMD (AOR = 1.0027, 95% CI = 1.0016, 1.0041). Although these associations were statistically significant, effect sizes were small.

## 4. Discussion

Environmental stressors in urban areas are associated with negative health risks including poor mental health [3,9]. Urban green land cover provides natural scenery and places for restoration from stress and mental fatigue [46,47,48,49]. This study examined the relationships between mental health and characteristics of urban green land cover measured by the amount of green cover (e.g., percent cover) and landscape patterns in 276 counties across the conterminous United States. Confounding factors associated with both urbanization and mental health were also considered. Results showed that longer distance between forest patches, suggestive of more spatially dispersed forests, had the most positive bivariate association with lower prevalence of frequent mental distress (FMD). In multivariable models, forest edge contrast index which indicates more connections between forest and built features was associated with lower mental distress.

Past research demonstrated that access to green space and viewing natural scenery in urban environments are associated with positive emotions, emotional fulfillment, and stress restoration [8,14,46,50]. Even small patches of urban green land cover such as street trees have positive effects on mental health [14]. Surprisingly, more forest cover exhibited a positive relationship with mental distress though it was not significant. Previous research reported a U-shaped dose-response curve for tree density and mental restoration. Jiang et al. reported tree density between 1.7% to 24% contributed to improved stress recovery and the recovery time started to decline when tree density was above 34% [51]. It is possible that there is a threshold for a given amount of landscape characteristics for improving mental health. Findings from the present study also revealed that other characteristics of green land cover (e.g., edge contrast index and patch cohesion index) might aid our understanding of the associations between green land cover and mental health status than percent land cover alone. This can be attributed to the role of edge fragmentation in making green spaces more accessible and supportive of recreational use and physical activity [52]. This study also indicates that different types of green land cover may have different effects on mental health. For example, previous research focused on New York City reported that tree cover was positively associated with reported good or excellent health status but not grass density [53]. Our findings showed that more aggregated shrubland contributed to increased odds of high levels of FMD. These findings suggest that not all the green land cover types have the same effects on health outcomes. Co-benefits associated with different land covers such as temperature and pollution mitigation and scenic views may also be critical for certain health outcomes [54].

Several studies reported that socioeconomic status was associated with mental health [43,55], as well as the process of urbanization [56,57]. Based on the bivariate results, higher median household income was significantly associated with lower mental distress. However, the strength of the land cover-FMD association attenuated slightly after adjusting for income. It is possible that other factors, particularly social engagement, are key mediators in green space-mental health relationships [1,5,58]. Current theorizing suggests that public green space may affect mental health through a number of pathways including social cohesion defined by shared values, norms and social ties in a neighborhood [5,6]. Significant associations between region and FMD were consistent with previous findings related to geographic variation in mental health [29,45].

Several limitations of this study exist. First, the cross-sectional analysis does not permit tests of causal relationships between land cover and mental health [5,59]. Future research should perform longitudinal studies and other appropriate designs to examine causal linkages. Second, the mental health measure relied on self-report and consisted of a single survey item. Third, the analyses relied on 30-m land cover data which compromises accurate land cover classification in densely developed urban areas. Higher resolution land cover data are needed for better identifying small and fragmented (e.g., street trees) green land cover in urban areas. Next, green land cover across the U.S. varies by climatic conditions and geographic features. For example, green land cover in southwestern U.S. counties included in this study are mostly shrubland, whereas in the northwest it is mostly forest. Therefore, greater specification of these regional differences offers opportunities for future research. Further studies should examine green land cover and health using a stratified approach. For example, separate models can be estimated by region or land cover types. Small effect sizes were also observed. In this regard, we acknowledge that mental distress arises from complex interactions of biological, psychosocial, and environmental factors [60]. Additional investigations should clarify pathways that link urban landscape characteristics to mental health. Strengths of the study include use of measures from a widely used and validated surveillance system and objective measures from a national land cover database. Furthermore, the study examined mental health in relation to several landscape metrics in addition to total land cover. With increasing population growth in urban areas and potential threats to green land cover, further research is needed to advance understanding of relationships between urban green land cover and public health. Our study aimed to provide direction for future research by highlighting the importance of studying both land cover classifications and specific landscape metrics in relation to mental health in urban areas.

## Figures and Tables

**Table 1:** Description of landscape metrics.

Variable	Description
Percent Land Cover	The proportional abundance of vegetative cover in the defined area (e.g., county).
Patch Area	Mean of the total area of vegetative patches.
Patch Density	The number of vegetative patches per 100 hectares.
Edge Density	The total length of all vegetative edge segments per hectare.
Edge Contrast Index	The total length of segments between vegetative cover and developed area divided by the total perimeter of all the patches.
Euclidean Distance	Mean distance to the nearest neighbor patch with the same vegetative type (based on shortest edge-to-edge distance).
Patch Cohesion Index	The physical connectedness of vegetative cover. The value increases as the patch type becomes more physically connected.

**Table 2:** Descriptive statistics for land cover classes, landscape metrics and potential confounding variables ^a^.

Variables	Mean	SD
Frequent mental distress	10.76	2.0
Forest	0.31	22.29
Patch area	8594.68	24,802
Patch density	1.54	0.9
Edge density	49.62	26.14
Edge contrast index	27.19	20.78
Euclidean distance	143.94	155.27
Patch cohesion index	96.58	4.98
Shrubland	5.94	13.04
Patch area (ha)	10,680.60	78,921.10
Patch density	1.18	1.22
Edge density	16.86	24.44
Edge contrast index	12.36	11.10
Euclidean distance	803.69	2058.48
Patch cohesion index	81.2	10.25
Herbaceous	6.66	11.95
Patch area (ha)	1254.51	10,091.60
Patch density	1.36	1.14
Edge density	18.63	20.73
Edge contrast index	17.84	10.9
Euclidean distance	328.89	448.87
Patch cohesion index	85.94	7.45
Potential Confounders		
Population density	402,639	823,130
Housing density	165,658	313,323
Median HH income	57,410 USD	13,336.70 USD
Race (% non-white population)	27.7	19.39

^a^ Units of measure for each variable were as follows: Land cover type (%), patch area (ha), patch density (#/100ha), edge density (m/ha), edge contrast index (%), Euclidean distance (m), patch cohesion index (%), population density (#/km^2^), housing density (#km^2^).

**Table 3:** Mean comparisons of Frequent Mental Distress by region.

Region	N (Counties)	Mean	SD
Southwest	40	9.5 ^a^	1.1
West	35	9.8 ^a^	1.2
Northeast	34	10.5 ^a,b^	1.2
Midwest	61	10.6 ^b,c^	1.2
Southeast	106	11.7 ^d^	1.4

ANOVA F = 26.15, df = 4271, *p* < 0.00001. ^a,b,c,d^ Mean values with different superscripts are statistically significant different at *p* < 0.01 (Scheffe Test).

**Table 4:** Spearman correlations of frequent mental distress (FMD) with land cover classes, landscape metrics and potential confounding variables.

Landscape Metric	rho	*p*-Value
Forest	0.18	0.003
Patch area	0.17	0.005
Patch density	−0.14	0.017
Edge density	0.11	0.059
Edge contrast index	0.05	0.386
Euclidean distance	−0.18	0.003
Patch cohesion index	0.17	0.004
Shrubland	−0.16	0.007
Patch area	−0.10	0.094
Patch density	−0.17	0.005
Edge density	−0.17	0.004
Edge contrast index	−0.08	0.188
Euclidean distance	0.19	0.001
Patch cohesion index	−0.08	0.175
Herbaceous	−0.14	0.019
Patch area	−0.11	0.070
Patch density	−0.03	0.670
Edge density	−0.14	0.025
Edge contrast index	−0.09	0.123
Euclidean distance	0.11	0.067
Patch cohesion index	−0.13	0.027
Potential Confounders		
Population density	−0.08	0.214
Housing density	−0.07	0.251
Median Household Income	−0.12	0.042
Race (% non-white population)	−0.14	0.016

Units of measure were as follows: Land cover type (%), patch area (ha), patch density (#/100ha), edge density (m/ha), edge contrast index (%), Euclidean distance (m), patch cohesion index (%), population density (#/km^2^), housing density (#km^2^).

**Table 5:** Logistic regression models of FMD adjusting for region, population and housing density, household income, and race.

		Adjusted Odds Ratio		95% CI	95% CI
				Lower CI	Lower CI
(Intercept)		0.0016	**	0.0000	0.0000
%Forest		1.0132		0.9917	0.9917
Forest-Edge contrast index		0.9619	**	0.9371	0.9371
Shrubland-Patch area		1.0000		1.0000	1.0000
Shrubland-Edge contrast index		1.0068		0.9595	0.9595
Shrubland-Euclidean distance		1.0027	***	1.0016	1.0016
Shrubland-Cohesion index		1.0751	**	1.0194	1.0194
%Herbaceous		0.9957		0.9347	0.9347
Herbaceous-Patch density		1.2880		0.8612	0.8612
Ecoregion	Midwest			Referent	
	Northeast	1.0288		0.3255	3.2753
	Southeast	1.9631		0.6698	5.8683
	Southwest	0.0088	***	0.0009	0.0666
	West	0.0453	**	0.0053	0.3155
Population Density				*DL*	
Housing Density		0.9990		0.9965	1.0014
Median Household Income		1.0000		1.0000	1.0000
%Non-White		1.0330	*	1.0063	1.0623

Pseudo-R^2^ (McFadden) = 0.365; Significance level: * indicates *p* <0.05, ** indicates *p* < 0.01, and *** indicates *p* < 0.001; DL: deleted from the model due to a GVIF > 2.
